# Reply to Liu et al.: Specific mutations matter in specificity and catalysis in ACE2

**DOI:** 10.1073/pnas.2024450118

**Published:** 2021-04-08

**Authors:** Jeff Glasgow, Anum Glasgow, Tanja Kortemme, James A. Wells

**Affiliations:** ^a^Department of Pharmaceutical Chemistry, University of California, San Francisco, CA 94158;; ^b^Department of Bioengineering and Therapeutic Sciences, University of California, San Francisco, CA 94158

In response to our recent publication describing affinity-enhanced, long−half-life ACE2-based receptor traps for severe acute respiratory syndrome coronavirus 2 (SARS-CoV-2) neutralization (1), Liu et al. (2) point to their parallel work showing substrate-dependent peptidase activity of ACE2 active site mutants (3). Understanding the physiologically relevant ACE2 peptidase activity determinants is critical, as several groups are developing ACE2-based receptor traps with intact (4, 5), modestly attenuated (6), or ablated activity (1, 7, 8) to separate the effects of blocking on angiotensin II (Ang II) conversion. Toward this end, we introduced an H345L mutation that is postulated to form part of the oxyanion binding site (9); mutations in the oxyanion hole for zinc metalloproteases (10) are well known to disrupt the tetrahedral oxyanion in the transition state and dramatically reduce activity. Indeed, the H345L mutation was reported by others (9) and us here to reduce activity in ACE2 over 300-fold against a commonly used fluorogenic peptide mimic (Dnp-APK(Mca)) as a proxy for ACE2 activity. Here, Liu et al. (2) verify that another mutant at this same site, H345A, was similarly inactive on Dnp-APK(Mca) but, interestingly, retained or even enhanced activity on Ang II. The authors conclude, without testing, that the H345L mutation we constructed retains activity toward Ang II.

**Fig. 1.**
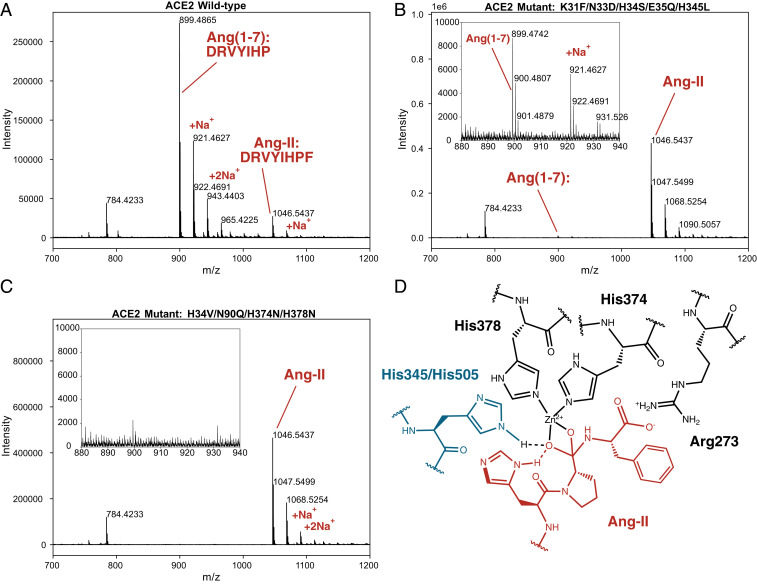
Characterization of Ang II hydrolysis by ACE2 variants using mass spectrometry. (*A*) CVD208 (wild-type ACE2(740)-Fc) (1 µg/mL) hydrolyzed >90% of Ang II (200 µM) over 60 min at 37 °C. (*B*) CVD313 (ACE2(740)-Fc (K31F/N33D/H34S/E35Q/H345L) and (*C*) CVD118 (ACE2(614)-Fc, H34V/N90Q/H374N/H378N) show virtually no detectable hydrolysis of Ang II under the same conditions. *Insets* in *B* and *C* show zoomed-in region containing Ang(1–7), with very little formation from CVD313. (*D*) Potential substrate-assisted catalysis in ACE2 activity. Based on Guy et al. (9), likely ACE2 residues for oxyanion stabilization include His345 or His505, shown in blue. His6 of Ang II may participate in substrate-assisted catalysis. Three C-terminal residues of Ang II are depicted in red.

We therefore tested our lead ACE2 receptor trap, CVD313 (K31F/N33D/H34S/E35Q/H345L), alongside controls including CVD208 (wild-type ACE2(740)-Fc), and a Zn^2+^-binding ablated mutant CVD118 (ACE2(614)-Fc-H34V/N90Q/H374N/H378N) for hydrolysis of Ang II. Contrary to the Liu et al. (2) expectation, we find that CVD313 has very little activity against Ang II (Fig. 1). For example, under conditions where the wild-type CVD208 cleaved >90% of Ang II (Fig. 1*A*) to yield the cleavage product Ang(1–7), for CVD313, we observed vanishingly small (<1%) levels of Ang(1–7) (Fig. 1*B*). We were also unable to observe formation of any Ang(1–7) using CVD118, confirming the necessity of Zn^2+^ in the active site (Fig. 1*C*).

What could account for the interesting differences in substrate-dependent activity for the H345L (inactive on both substrates) versus H345A (inactive on Dnp-APK(Mca) but active on Ang II)? It is possible the differences depend on the other mutations in CVD313. Alternatively, it is striking to note that Ang II contains a P2 His residue not found in Dnp-APK(Mca); it is possible that the P2 His cooperates with H345 to form part of the oxyanion binding (Fig. 1*D*). Some years ago, our group discovered substrate-assisted catalysis where mutation of the catalytic H64A in subtilisin reduced activity by 10^6^ against standard substrates, but was substantially restored by substrates containing a P2 His which can bind in the cavity created by H64A and participate in catalysis (11). By this model in ACE2, a bulky H345L mutation would be far more disruptive than H345A. Thus, we thank Liu et al. (2) for presenting their H345A result which, alongside ours, could reveal insights into ACE2 mechanism and substrate specificity.
